# Benzodiazepine Use Disorder Observed and Diagnosed in a Tertiary Care Pediatric Specialty Clinic: A Descriptive Retrospective Chart Review

**DOI:** 10.1192/bjo.2024.211

**Published:** 2024-08-01

**Authors:** Alex Smithers, Laura Miller, Melanie MacInnis, Selene Etches

**Affiliations:** 1 NS Health, Halifax, NS, Canada; 2 IWK Health, Halifax, NS, Canada

## Abstract

**Aims:**

**Objectives:** In youth and young adults, it is common to encounter non-medical use of benzodiazepines, defined as use without a prescription or use for reasons other than that for which the medication is intended. Benzodiazepine use disorder remains understudied and overlooked, especially in youth and young adults. The primary objective of our study was to highlight the proportion of youth and young adults with aberrant use of benzodiazepines and diagnosed with benzodiazepine use disorder in a single centre. The secondary objective was to determine factors associated with aberrant benzodiazepine use and benzodiazepine use disorder in that sample.

**Methods:**

This retrospective chart review screened for benzodiazepine use in 310 adolescent patients aged 12–19 seen for the first time in a concurrent disorders clinic, at a tertiary care clinic in Canada. Of those 310 patients, 167 were included in the final chart review.

**Results:**

97.6% of patients who used benzodiazepines demonstrated aberrant use, and 39.3% of patients received a diagnosis of benzodiazepine use disorder.

**Conclusion:**

This review showed that a substantial percentage of youth and young adults in a concurrent disorders clinic in Canada are presenting with aberrant benzodiazepine use and are being diagnosed with benzodiazepine use disorder. Despite this prevalence, there is little by way of literature to guide treatment of benzodiazepine use disorder in this population.

